# Short‐term clinical outcomes of laparoscopic gastrectomy for remnant gastric cancer: A single‐institution experience and systematic review of the literature

**DOI:** 10.1002/ags3.12221

**Published:** 2018-11-03

**Authors:** Ryota Otsuka, Hideki Hayashi, Haruhito Sakata, Masaya Uesato, Koichi Hayano, Kentaro Murakami, Masayuki Kano, Takeshi Fujishiro, Takeshi Toyozumi, Yoshihide Semba, Hisahiro Matsubara

**Affiliations:** ^1^ Department of Frontier Surgery Graduate School of Medicine Chiba University Chiba Japan

**Keywords:** gastrectomy, laparoscopic, open gastrectomy, remnant gastric cancer, systematic review

## Abstract

**Aim:**

Application of laparoscopic approaches for the treatment of remnant gastric cancers (RGC) is still controversial. Therefore, in the present study, the safety and effectiveness of laparoscopic gastrectomy (LG) for RGC was investigated.

**Methods:**

A total of 27 patients who underwent gastrectomy for RGC from June 2008 to September 2017 were enrolled in this study. A comprehensive review of the literature on LG for RGC published before December 2017 using the PubMed database was carried out.

**Results:**

Laparoscopic gastrectomy was carried out in seven patients, and open gastrectomy (OG) was done in the remaining 20 patients. LG was associated with significantly less intraoperative blood loss (70 ± 71 vs. 1066 ± 1428 g; *P* < 0.001), significantly more retrieved lymph nodes (22 ± 13 vs. 12 ± 9; *P* = 0.03), a relatively lower postoperative complication rate, and a relatively shorter postoperative hospital stay than OG. A comprehensive review of the literature showed that LG for RGC was more likely to correlate with longer operative time, less blood loss, lower postoperative complication rate, shorter postoperative hospital stay, and more retrieved lymph nodes than OG.

**Conclusion:**

The clinical outcome of our patients with RGC and the literature indicated that a laparoscopic approach contributed to faster recovery after surgery than an open approach without sacrificing its radicality and was a safe and secure treatment option for RGC.

## 1 INTRODUCTION

Remnant gastric cancers (RGC) were initially defined as gastric cancer that occurs after gastrectomy for benign disease.^1^ It now represents all cancers developed in the remnant stomach, regardless of the malignancy of the initial disease.^2^, ^3^, ^4^

Although chemotherapy and radiotherapy have been developed in the field of treatment for gastric cancer, gastrectomy with adequate lymphadenectomy remains the first treatment option for resectable gastric cancers.^5^, ^6^ With the expansive application of laparoscopic surgeries for gastric cancer, the first case of laparoscopic gastrectomy (LG) for RGC was reported by Yamada et al in 2005.^7^ Several other reports with similar LG for RGC have followed since then. However, the number of cases reported has remained small due to the rarity of RGC,^8^ and the usability of laparoscopic approaches for the treatment of RGC is still unclear.

Therefore, the purpose of the current study was to investigate the safety and effectiveness of LG for RGC. To that end, we compared short‐term clinical outcomes of LG for RGC with those of open gastrectomy (OG) in our institute and reviewed all published English language literature on LG for RGC.

## 2 MATERIALS AND METHODS

### 2.1 Patients

A total of 27 patients who underwent gastrectomy for RGC at Chiba University Hospital from June 2008 to September 2017 were enrolled in this study. LG was carried out in seven patients, and OG was done in the remaining 20 patients. Stage of the disease was determined according to the TNM classification (UICC 8th edition),^9^ and the severity of the postoperative complications was estimated according to the Clavien‐Dindo classification.^10^

This study was in agreement with the guidelines of the institutional ethics committee and was conducted in accordance with the Declaration of Helsinki.

### 2.2 Surgical procedures

Indication for a laparoscopic procedure for primary gastric cancer at our institution is a preoperative diagnosis of stage 1A to stage 2A disease. As such, the same criterion was applied to the patients with RGC.

Under general anesthesia, the patient was placed in the supine position with the legs apart. Then, a 12‐mm camera port was placed in either the umbilical or left subcostal area by the open method. CO_2_ insufflation was maintained at 10 mm Hg, and four working ports were placed, as described previously.^11^, ^12^ Adhesiolysis was carried out with one or two working ports if needed for insertion of the other trocar. Dissection around the previous anastomosis was carried out, and the duodenum was divided at the immediate anal side of the anastomosis. Additional lymphadenectomy was done according to the recommendations of the guidelines,^6^ with the corresponding tumor location of the original stomach, if necessary. After the resected specimen was removed, continuity of the alimentary tract was resumed intracorporeally with Roux‐en‐Y reconstruction.

### 2.3 Literature review

We explored the PubMed database for English language case reports, case series, and case comparative studies of LG for RGC published before December 2017 using the following key words: “remnant gastric cancer” OR “gastric remnant cancer” OR “gastric stump cancer” AND “gastrectomy” AND “laparoscopy.” Related citations of all relevant articles were assessed to identify other related reports.

### 2.4 Statistical analyses

All statistical analyses were carried out using the IBM SPSS Statistics, version 21 (IBM Corp., Armonk, NY, USA). Categorical variables were assessed using Fisher's exact test. Continuous variables were evaluated using Wilcoxon's rank‐sum test or Student's *t* test or Welch's *t* test, according to the data distributions. Statistical significance was defined as a *P* value < 0.05.

## 3 RESULTS

### 3.1 Patient characteristics

Clinicopathological features of the patients are summarized in Table 1. Mean age of the patients was 73.3 ± 8.2 years, and the male‐to‐female ratio was 25:2. Mean time interval from the previous surgery was 29.8 ± 18.5 years, and the benign‐to‐malignant ratio was 16:11. In the initial surgery, 24 cases underwent an open procedure, and three underwent a laparoscopic procedure. Most cases were reconstructed with Billroth I (13 cases) or II (13 cases). For the treatment of RGC, laparoscopic total gastrectomy was carried out in five cases and resection of the distal part of the remnant stomach in two, without any conversion to open surgery. Open total gastrectomy was chosen for the other 20 cases.

**Table 1:** Characteristics of patients who underwent gastrectomy for RGC from June 2008 to September 2017

	N = 27
Age, y (range)	73.3 (58‐88)
Gender (male/female)	25/2
BMI (kg/m^2^)	21.2 ± 3.2
Time interval (y)	29.8 ± 18.5
Previous disease	
Benign/Malignant	16/11
Previous surgical approach	
Open/Laparoscopy	24/3
Previous operation	
DG‐B1	13
DG‐B2	13
DG‐RY	1
Surgical procedure	
LTG/LDG/TG	5/2/20

B1, Billroth I; B2, Billroth II; BMI, body mass index; DG, distal gastrectomy; LDG, laparoscopic distal gastrectomy; LTG, laparoscopic total gastrectomy; RY, Roux‐en‐Y; TG, total gastrectomy.

### 3.2 Clinical outcomes of our series

Basic characteristics, such as age, gender, body mass index (BMI), previous disease type, previous surgical approach, and time interval of operations, were compared between the LG and OG groups, with no statistically significant differences noted (Table 2). Regarding the surgical outcome, we compared operative time, blood loss, and number of retrieved lymph nodes between the groups (Table 2), with no statistically significant inferiority of LG noted compared with OG. Conversely, there was significantly less blood loss in the LG group than in the OG group (70 ± 71 vs. 1066 ± 1428 g, respectively; *P* < 0.001), and the number of retrieved lymph nodes was significantly larger in the LG group than in the OG group (22 ± 13 vs. 12 ± 9, respectively; *P* = 0.03) (Table 2).

**Table 2:** Clinical background and surgical outcome of patients according to surgical approach

Characteristic	LG n = 7	OG n = 20	*P* value
Age (y)	71.3 ± 9.7	74.1 ± 7.7	0.45
Gender			
Male	7	18	1.0
Female	0	2	
BMI (kg/m^2^)	22.1 ± 2.5	20.9 ± 3.4	0.41
Time interval (y)	39 ± 22	26 ± 16	0.12
Previous disease			
Benign	5	11	0.66
Malignant	2	9	
Previous surgical approach			
Open	6	18	1.0
Laparoscopy	1	2	
Operative time (min)	364 ± 95	309 ± 104	0.23
Blood loss (g)	70 ± 71	1066 ± 1428	<0.001
Retrieved lymph node	22 ± 13	12 ± 9	0.03
Other organ resection	1	3	1.0
Final stage			
I/II/III/IV	6/1/0/0	12/5/3/0	0.90

BMI, body mass index; LG, laparoscopic gastrectomy; OG, open gastrectomy.

Postoperative course was also compared between the groups. The OG group had three patients with T4 disease, which exceeded our indications for laparoscopic procedures, and a benign previous disease type was significantly associated with more retrieved lymph nodes (19.5 ± 10.4 vs. 7.2 ± 6.9, respectively; *P* = 0.002) than a malignant type. Therefore, we excluded the patients with T4 disease and divided the remaining patients into two subgroups according to previous type of disease. We then compared the postoperative outcomes between these two subgroups (Table 3). The background characteristics and surgical outcomes of the patients in this analysis were similar to the former results except for the age in the subgroup with a malignant previous disease type (patients in the LG group were significantly younger than those in the OG group). Regarding the postoperative course, a relatively low incidence of complications and a relatively short postoperative hospital stay in the LG group were noted in both subgroups, but the differences were not statistically significant (Table 3). No mortality was observed in either group.

**Table 3:** Clinical background and surgical outcome of patients according to previous type of disease

Previous disease type	Benign			Malignant		
Surgical approach	LG	OG	*P* value	LG	OG	*P* value
No. of cases	5	10		2	7	
Age	75.8 ± 7.2	75.8 ± 7.3	0.40	60 ± 2.8	75.4 ± 4.4	0.03
Sex (Male/Female)	5/0	8/2	0.52	2/0	7/0	–
BMI (kg/m^2^)	21.7 ± 2.8	22.6 ± 3.7	0.62	23 ± 1.7	19.7 ± 2.2	0.09
Operative time (min)	379 ± 109	293 ± 120	0.20	326 ± 49	295 ± 72	0.60
Blood loss (g)	78 ± 78	862 ± 860	0.001	50 ± 71	529 ± 309	0.06
Retrieved lymph node	24 ± 14	18 ± 9	0.26	16 ± 9	4 ± 3	0.33
Final stage			0.58			–
I/II/III/IV	4/1/0/0	5/5/0/0		2/0/0/0	7/0/0/0	
Complications			0.28			0.58
Moderate (Grade I or II)	2	1		0	1	
Severe (Grade ≥IIIa)	0	3		0	3	
Postoperative hospital stay (d)	14 ± 6	25 ± 21	0.27	12 ± 1	26 ± 14	0.24

BMI, body mass index; LG, laparoscopic gastrectomy; OG, open gastrectomy.

### 3.3 Review of the literature

Six case‐control studies, seven case series and three case reports on LG for RGC were retrieved by our literature search (Table 4).^2^, ^3^, ^4^, ^7^, ^13^, ^14^, ^15^, ^16^, ^17^, ^18^, ^19^, ^20^, ^21^, ^22^, ^23^, ^24^ One institution reported a case‐control study^2^ after the publication of a case series.^25^ Therefore, the latter case series was excluded from the analysis to avoid double inclusion of the cases.

**Table 4:** Summary of the latest case series of laparoscopic gastrectomy for remnant gastric cancer

Author, Year, Country	Approach	n	Conversion to OG	Operative time (min)	Blood loss (g)	Retrieved lymph node	Postoperative hospital stay (d)	Complications
Yamada et al 2005, Japan^7^	LG	1	None	274	30	NA	NA	None
Corcione et al 2008, Italy^13^	LG	3	None	210	Minimal	18	11	1/3 (33.3%)
Cho et al 2009, Korea^14^	LG	2	None	487.5 ± 74.2	425 ± 35.4	14.5 ± 7.8	NA	None
Qian et al 2010, China^15^	LG	15	1/15 (7%)	205 ± 25	110 ± 40	18 ± 5	NA	1/15 (7%)
Shinohara et al 2013, Japan^16^	LG	5	None	370.8 ± 114.4	63.6 ± 95.7	18.2 ± 5.1	8.8 ± 0.4	None
Pan et al 2014, China^19^	LG	3	None	251.7 ± 27.5	76.7 ± 25.2	16.7 ± 6.1	8 ± 1	None
Nagai et al 2014, Japan^18^	LG	12	None	362.3 ± 68.4	65.8 ± 62	23.7 ± 10.7	11.3 ± 2.8	None
	OG	10	NA	270.5 ± 94.9	746.3 ± 577.1	15.9 ± 7.6	24.9 ± 10	2/10 (20%)
Kwon et al 2014, Korea^2^	LG	18^a^	1/18 (5.6%)	266.2 ± 77.2	182.2 ± 188.7	8	6	6/18 (33.3%)
	OG	58	NA	203.3 ± 52.2	193.1 ± 227.6	7	9	26/58 (44.8%)
Kim et al 2014, Korea^17^	LG	17	None	197.2 ± 60.6	NA	NA	11.1 ± 8.7	4/17 (23.5%)
	OG	50	NA	149.3 ± 46.9	NA	NA	13.8 ± 9.4	15/50 (30%)
Kim & Kim 2015, Korea^20^	LG	1	None	200	100	24	13	None
Yamamoto et al 2015, Japan^23^	LG	3	None	356.7 ± 45.1	41.7 ± 20.2	26 ± 21.7	20.3 ± 1.5	None
Korehisa et al 2015, Japan^21^	LG	4	None	413.3 ± 52.1	270.3 ± 191.7	NA	18 ± 6.3	1/4 (25%)
Son et al 2015, Korea^3^	LG	17	8/17 (47.1%)	234.4 ± 65.2	227.6 ± 245	18.8 ± 12.3	9.3 ± 3.2	6/17 (35.2%)
	OG	17	NA	170 ± 39.5	184.1 ± 123.1	22.3 ± 14.4	9.3 ± 3.1	5/17 (29.4%)
Luo et al 2015, China^22^	LG	9	None	221.1 ± 19.5	105.6 ± 35.0	16.2 ± 3	NA	1/9 (11.1%)
	OG	9	NA	212.9 ± 14.3	147.8 ± 41.9	16.7 ± 3.3	NA	2/9 (22.2%)
Kim & Kim 2016, Korea^24^	LG	1	None	295	200	20	7	None
Tsunoda et al 2016, Japan^4^	LG	10	None	324.5 ± 42.8	55.4 ± 63.9	22.4 ± 15.8	12.5 ± 2.9	1/10 (10%)
	OG	6	NA	289	893	7	24	2/6 (33.3%)
Current study	LG	7	None	364 ± 95	70 ± 71	22 ± 13	13 ± 5	2/7 (28.6%)
	OG	20	NA	309 ± 104	1066 ± 1428	12 ± 9	27 ± 21	10/20 (50%)

LG, laparoscopic gastrectomy, NA, not applicable; OG, open gastrectomy.

a Ten laparoscopic, eight robotic.

In the 17 total studies, including our own, 128 patients underwent LG for RGC. Fourteen of the 17 studies showed no conversion to OG, and the other three reported a 5.6%‐47.1% conversion rate to OG.^2^, ^3^, ^15^ In all of the studies, mean operative time was 197‐487.5 minutes, mean blood loss was minimal ‐425 g, mean number of retrieved lymph nodes was 8‐26, and mean postoperative hospital stay ranged from 6 to 20.3 days. Eight studies showed no postoperative complications, but the other nine reported a postoperative complication rate ranging from 7% to 35.2%.

Seven studies, including our own, conducted comparative analyses between LG and OG for RGC. Although all studies showed a longer operative time for LG than for OG, a lower postoperative complication rate with LG was observed in six studies. Furthermore, blood loss was lower and postoperative hospital stay was shorter in five of the six studies, and more lymph nodes were retrieved with LG than with OG in four of the six studies.

## 4 Discussion

The aim of the present study was to examine the safety and effectiveness of LG for RGC. A comparative study of RGC in our institution indicated that LG was associated with significantly less intraoperative blood loss, significantly more retrieved lymph nodes, a relatively lower postoperative complication rate, and a relatively shorter postoperative hospital stay than OG, and these results were consistent with those of our comprehensive review of the literature.

Similar features have also been reported for primary laparoscopic gastric cancer surgeries. A laparoscopic approach provides a magnified bird's‐eye view in the surgical field. Visualization of the fine anatomy in the abdominal cavity, such as thin nerve bundles and vessels, could illuminate landmarks for optimal lymphadenectomy for surgeons and potentially help reveal more lymph nodes and reduce intraoperative blood loss.^26^ The lower incidence of postoperative complications indicated in six of the seven comparative studies may also be associated with this feature of laparoscopic surgeries and, similarly, our series indicated no severe postoperative complications and no mortality in the LG group.

In our study, a benign previous disease type was significantly associated with a longer interval between operations and more retrieved lymph nodes but not with operative time or blood loss (Table 3). Surgeries for malignant diseases potentially cause more adhesions in the surgical field than those for benign disease, but this factor does not likely affect the difficulty (ie operative time or blood loss) in surgeries for RGC, although, in our cases, relatively few patients had malignant previous disease.

In this context, LG has been proposed as a standard treatment for RGC. However, surgical treatments of patients with RGC may be more challenging than in primary cases, as patients with RGC may have adhesions and anatomical alterations caused by preceding surgeries. Furthermore, a laparoscopic approach requires a more advanced technique than an open approach. Our initial case of LG for RGC was carried out 13 years after laparoscope‐assisted surgery for early gastric cancer was started in our institute, and 4 years after the switch to totally laparoscopic procedures. Therefore, the aforementioned favorable data for LG may have been achieved only in institutions with adequate laparoscopic techniques.

Although several reports have described comparative case studies between LG and OG for RGC, the number of cases examined is small at present.^2^, ^3^, ^4^, ^17^, ^18^, ^22^ Only two reports have described long‐term clinical outcomes,^2^, ^18^ and both studies indicated comparable 5‐year survival rates between the LG and OG groups. However, these results remain inconclusive because of their short follow‐up time (25.2 and 39.1 months). Therefore, further comparative case studies with longer follow‐up periods are awaited to establish LG as a standard treatment option for RGC.

Several limitations associated with the current study should be mentioned. First, this study had a retrospective design, which prevented us from excluding potential selection biases. Therefore, a randomized controlled study should be considered to confirm the putative validity. Second, only a small number of patients could be enrolled in this study because of the rare incidence of the disease in question. We made up for this drawback by conducting a comprehensive review of all publications on LG for RGC to elucidate more reliable features of LG for RGC.

In summary, our analyses indicated that laparoscopic gastrectomy was associated with significantly less intraoperative blood loss, significantly more retrieved lymph nodes, a relatively lower postoperative complication rate, and a relatively shorter postoperative hospital stay than open gastrectomy. Therefore, a laparoscopic approach may be a safe and secure treatment option for remnant gastric cancer as well as for primary gastric cancers.

## Disclosure

This study was in agreement with the guidelines of the institutional ethics committee and was conducted in accordance with the Declaration of Helsinki.

Conflicts of interest: Authors declare no conflicts of interests for the present article.
